# Toxoplasmosis Immunity Status of Blood Donors in Sidi Bel Abbès, West Algeria

**DOI:** 10.7759/cureus.28826

**Published:** 2022-09-06

**Authors:** Malika Belkacemi, Benasissa Heddi

**Affiliations:** 1 Hemobiology and Blood Transfusion, Hassani Abdelkader University Hospital, Sidi Bel Abbès, DZA; 2 Faculty of Medicine, University Djillali Liabes, Sidi Bel Abbès, DZA; 3 Central Laboratory, Hassani Abdelkader University Hospital, Sidi Bel Abbès, DZA

**Keywords:** blood transfusion, risk of transfusion transmission, blood component, seroprevalence, blood donors, toxoplasma gondii, toxoplasmosis

## Abstract

Background

Toxoplasmosis is a zoonotic disease. It is due to an obligate intracellular protozoan called *Toxoplasma gondii (T. gondii*). Felids are considered definitive hosts, and humans take part as intermediate hosts. At least one-third of the world’s population is seropositive to the parasite. In addition, to the known modes of transmission, the infection can be transmitted through blood transfusions. The aim of this study is to assess the immune status of blood donors about this disease and estimate the potential risk by blood components.

Methodology

A single cross-sectional study was conducted based on the search for *T. gondii* antibodies (IgG and IgM) in blood donors. This research was performed using a latex particle agglutination assay confirmed by an enzyme-linked immunosorbent assay (ELISA).

Results

In all, 103 blood donors were involved in this study. The sex ratio of male/ female was 0.75. The recorded rate of exposure to toxoplasmosis in blood donors was 47.7% (95% CI: 35.1-54.3). Significant differences were observed between the prevalence and those of other African countries in West, East, and Central Africa, but not with those of Algerian pregnant women and neighboring North African countries. There was no association between *T. gondii *seropositivity and the following factors: sex, age, and blood group ABO or Rhesus. Antitoxoplasma IgG was detectable in all positive donors, while IgM was undetectable. All seropositive donors had an IgG titer ≥9 IU/ml. The potential risk of *T. gondii* transmission ranges from 1 per 100,000 to 17 per 100,000 blood donations.

Conclusion

The seroprevalence of *T. gondii* infection was comparable to those found in Algerian pregnant women and neighboring North African countries. However, the seroprevalence rate was lower than recorded in other African countries. There is even a risk of transmission of toxoplasmosis through blood transfusions. There is a need to enhance blood safety measures for pregnant, immunocompromised, and multi-transfused people. As the immune status of blood donors may vary by region, there is a need to extend the national studies to the entire country. This study provides the first data on the seroprevalence of *T. gondii* infection among Algerian blood donors and the risk of its transmission by transfusion of blood components.

## Introduction

Toxoplasmosis is a zoonotic disease. It occurs due to an obligate intracellular protozoan called *Toxoplasma gondii* (*T. gondii*). It is known to be one of humans' most common parasitic infections. Felids are definitive hosts, and humans take part as intermediate hosts. There are two forms of *T. gondii* in humans: Tachyzoite is the active proliferating form seen in the initial, more acute stage of the infection, and Bradyzoite is the slow dividing form. Bradyzoite was developed from tachyzoite. In tissues, bradyzoites form cysts due to the host immune response [1]. Tissue cysts are found in the brain and muscles. In the environment, there is a sporozoite form enclosed in an oocyst. Infection is acquired by ingesting uncooked or contaminated meat, food, or water and by getting in contact with cat or dog feces in the soil. It can also be passed from mother to fetus if a woman is infected during or just before pregnancy [2]. In addition to these known modes of transmission, it has been shown that the infection can be transmitted through blood transfusion or organ transplant from an infected donor [3]. The disease in immunocompetent humans is asymptomatic or with very few clinical manifestations. Still, the parasite may "hide," stay inactive for years, and reactivate when the immune system is compromised. The disease is a significant public health concern. It can have severe or fatal consequences for immunocompromised patients, transplant recipients, and fetuses.

Toxoplasmosis has been reported in almost every part of the world. About a third of the human population is seropositive to the parasite [3]. It is now well established from various studies that the frequency varies from region to region according to geographical areas, dietary habits, and transmission routes. In the case of Algeria, it has been recorded that the prevalence in pregnant women was 47.8% (95% CI: 44.8-51.0) [4]. So far, there is no data on the disease in blood donors in Algeria. Furthermore, there is no recommendation to screen blood donors for *T. gondii*. So, in Algerian blood banks, screening tests for infectious diseases do not contain *T. gondii*.

Moreover, this disease is not notifiable in Algeria. To our knowledge, seroprevalence in blood donors provides essential data on the prevalence of infection in the general population. Thus, this study aims to assess the immune status of blood donors and estimate the risk of toxoplasmosis transmission through blood components.

## Materials and methods

Selection of subjects and study design

We conducted a single cross-sectional study from October 2018 to December 2018. We carried it out at the blood transfusion department of Sidi Bel Abbès city. We invited regular blood donors who live in Sidi Bel Abbès city to participate and give their consent. The criteria for inclusion and non-inclusion were those of blood donors as recommended by the National guidelines of blood donor selection. We included in this study any Algerian adult male and female able to donate after the physical examination and routine questionnaire. The ethics committee of the University Hospital of Sidi Bel Abbès approved the study. The purpose and procedures of the study were explained to donors. Written informed consent was obtained from all of them. The population consists of about 606 regular donors. The sample size was obtained by using a sample size calculator (https://www.calculator.net/sample-size-calculator.html). For these purposes, we considered a 95% CI, a 5% error, and a 6% prevalence for regular blood donors. Therefore, the minimum sample size needed for this survey was 76 donors.

Donor sampling and processing

About 5 mL of venous blood was collected from each blood donor. The serum was separated from the whole blood by centrifugation at 3500 x g for 15 min. The labeled tubes of serums were frozen and stored at -30∘C until analysis.

Assay

All samples were screened for *T. gondii* antibodies by latex particle agglutination using Pastorex™ Toxo, Biorad, France. All positive results were tested by enzyme-linked immunosorbent assay (ELISA). We used both ELISA kits. Platelia™ Toxo IgM Biorad kit was used to qualitatively detect IgM antibodies. Platelia™ Toxo IgG Biorad kit for the quantitative determination of IgG antibodies. The test results for IgG were interpreted as recommended by the manufacturer. Titer of IgG were <6 I.U./ml, negative; 6-9 I.U./ml, equivocal; and >9 I.U./ml, positive. All tests were performed following the instructions of the manufacturer.

Risk assessment

To estimate the risk of contamination of a blood donation by *T. gondii*, we used the mathematical model of the French Institute for Public Health Surveillance. It was estimated by taking the incidence in Algerian pregnant women. The risk is equal to the probability of taking a blood donor during parasitemia multiplied by the incidence of infection. The probability of taking such donors ranges from one to 21 days out of 365 days, with an incidence of 0.3 % [5].

Data analysis

The Kolmogorov-Smirnov test of normality was used to analyze the values. Continuous variables are expressed as the mean and SD or the median and range in the case of not normally distributed data. Paired and unpaired data were compared using the Wilcoxon and U-Whitney tests. A t-test was used to compare the means between groups. Besides, qualitative variables are reported as numbers or percentages with 95% CIs. Chi-square or Fisher's test was used to check the association of seropositivity with transfusion data. The ORs and their 95% CIs were calculated. Data were analyzed using IBM SPSS Statistics software. For all statistical tests, p<0.05 was considered statistically significant.

## Results

A total of 103 blood donors were enrolled in this study. The sex ratio of male/female was 0.75. Baseline data for all patients, men and women, are shown in Table 1, where we can observe a similar distribution between genders.

**Table 1:** Baseline data of 103 qualified blood donors. Chi-square test and *Mann-Whitney U test. Data are expressed as median value (minimum-maximum). NS: Nonsignificant difference.

	All	Male	Female	P-value
Number of subjects	103	44	59	
Age (years)	31 (19-61)	32.5 (20-49)	31 (19-61)	NS*
Blood Group ABO				NS
O	44	18	26	
A	30	14	16	
B	22	9	13	
AB	7	3	4	
RH Positive/RH Negative	84/19	33/11	51/8	NS
Anti-Toxoplasma gondii antibodies				NS
IgG Negative/IgG positive	57/46	24/20	33/26	
IgM Negative/IgM positive	103/0	44/0	59/0	

The prevalence of exposure to toxoplasmosis in blood donors was 47.7%. (95% CI: 35.1-54.3). The comparison of the prevalence of the current study and other reports is summarized in Table 2. It is apparent from this table that the prevalence found in this study was not significantly different from those found among pregnant women in Algeria and neighboring North African countries. However, significant differences were observed between the prevalence and those of other African countries in West, East, and Central Africa, with 0.05 > p>0.01 and p<0.001. 

**Table 2:** Seroprevalence of toxoplasmosis from reports of neighboring countries. ELISA: Enzyme-linked immunosorbent assay; EIA: Enzyme immuno assay; IFA: Immuno fluorescence assay; NS: Nonsignificant difference.

Author	Country	Population	Number	Method	Prevalence % (95% CI)	P-value	Reference
Belkacemi M and Heddi B (2022)	Algeria	Blood donors	103	ELISA	44.7 (35.1-54.3)		Current Study
Messerer L et al. (2014)	Algeria	Pregnant women	1028	EIA	47.80 (44.80-51.00)	NS	[5]
Lachkhem A et al. (2020)	Tunisia	Blood donors	800	ELISA-IFA	44.40 (40.93-47.82)	NS	[6]
Laboudi M (2014)	Morocco	Pregnant women	1169	ELISA	47.00 (46.97-47.03)	NS	[7]
Mousa DA et al. (2011)	Libya	Pregnant women	143	ELISA	44.80 (44.72-44.88)	NS	[8]
Elsheikha HM et al. (2004)	Egypt	Blood donors	260	ELISA	59.60 (95.54-59.66)	NS	[9]
Siransy L et al. (2016)	Ivory Coast	Blood donors	106	ELISA	67.92 (67.83-68.01)	= 0.0210	[10]
Abamecha F and Awel H (2016)	Ethiopia	Pregnant women	232	ELISA	85.30 (85.35-85.25)	<0.0001	[11]
Doudou Y et al. (2014)	Congo	Pregnant women	781	ELISA	80.30 (77.50-83.10)	<0.0001	[12]

As Table 3 shows, there is no association between *T. gondii* seropositivity and the following factors: sex, age, and blood group ABO or Rhesus.

**Table 3:** Factors related to blood donors associated with toxoplasmosis. NS: Nonsignficant difference.

Variable	Anti-Toxoplasma	T. gondii Antibodies	P-value
	Positive	Negative	
Age (years)	31.5 (19-58)	31 (19-61)	NS
Sex			NS
Male	20	24	
Female	26	33	
Blood Group			NS
O	21	23	
A	12	18	
B	10	12	
AB	3	4	
RH Blood Group			NS
RH Positive	38	46	
RH Negative	8	11	
Total	46	57	

**Figure 1:**
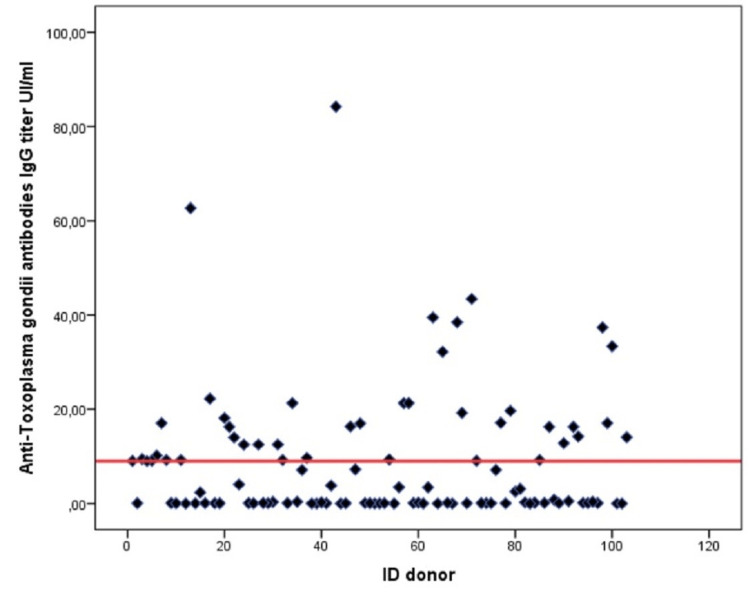
Distribution of anti-Toxoplasma gondii antibodies IgG titer among blood donors.

Antitoxoplasma IgG was detectable in all positive donors, while IgM was undetectable. From the graph below, shown in Figure 1, we can see that all seropositive donors had an IgG titer ranging from 9 to 84 IU/ml.

Table 4 presents a quantitative assessment of the risk of contamination of a blood donation by *T. gondii* in an endemic situation. A closer look at this table shows that the risk of transfusion transmission of toxoplasmosis ranges from 1 per 100,000 to 17 per 100,000 for donations.

**Table 4:** The quantitative estimation of the risk of contamination of a blood donation by Toxoplasma gondii.

Items	Low hypothesis	High hypothesis
Number of days	365	365
Span of parasitemia (days)	1	21
Probability to take a blood donor in parasitemia stage	1/365	21/365
Incidence of infection %	0.3	0.3
Risk of infected donation for 100,000 blood donations	1	17

## Discussion

This study permitted us to estimate the seroprevalence of toxoplasmosis in blood donors. The prevalence rate obtained in this study is in line with that found in Algerian pregnant women [5]. Moreover, this prevalence was comparable to those described in neighboring countries in North Africa due to the same socio-cultural behaviors [6-9]. However, the prevalence was lower than recorded in other African countries of West, East and Central Africa [10-12]. This discrepancy in prevalence between these countries may be ascribed to differences in economic development levels, hygienic conditions, and feeding habits, such as eating raw meat in Ethiopia [13]. Another reason for this difference may be linked to the local climatic conditions. It is well known that T. gondii is widely spread, mainly in warm and humid areas [14]. It has been shown that at temperate to tropical temperatures, oocysts remain infectious for up to one year [2].

It is notable that there was no link between sex and the seropositivity rate. Thus, both genders are at equal risk of exposure to toxoplasmosis. This observation is in accord with the available data [9,15]. Furthermore, we found no association between age and the presence of T. gondii antibodies. However, some studies describe an increase in seroprevalence with age due to the increased risk of being exposed to infection sources [16,17]. Therefore, our result may be explained by the fact that most of our blood donors are young. Moreover, no association was found between the ABO or RhD blood group and the presence or absence of T. gondii antibodies. These findings support evidence that these blood groups are not a crucial factor in high or reduced risk of infection [18,19].

Consistent with the literature, this study showed that blood donors had the infection in the past, which may be occurred at a younger age [19-21]. It is well known that a hallmark of toxoplasmosis is that the parasite persists for months to years or even a lifetime in tissues. Chronic disease underlies recrudescent ocular toxoplasmosis in immunocompetent individuals. It has been associated with several psychiatric disorders and behavioral changes [22]. This finding implies that the blood donors are exposed to retinochoroiditis, encephalitis, or epilepsy later in life. In some cases, it has been observed that latent toxoplasmosis can reactivate and become acute in blood [23]. Thus, the active disease may lead to the possibility of a hematogenous transmission. It has been shown that T. gondii was isolated from the blood of infected donors up to four years after infection [24]. Hence, the presence of the parasite in the bloodstream can be a source of infection for patients receiving a blood transfusion. It has been demonstrated that latent toxoplasmosis is correlated with various disease burdens. Thus, we think it is a significant health issue in our area [25].

The key finding to emerge from the analysis is that there is a risk of transfusion-transmissible toxoplasmosis in our region. So, the danger of transmission of T. gondii infection by receiving a blood transfusion from apparently healthy asymptomatic persons is of great societal concern. However, this risk has been neglected. This fact may explain that screening for T. gondii infection is not currently performed in blood banks. Moreover, It is well known that parasites can survive in leukocytes for up to seven weeks between 2 °C and 4 °C [26]. This ability has been observed to be a factor that increases the risk of transmission through blood transfusion [27]. As far we know, not all blood products are routinely leukoreduced in our blood bank. Thus, it can pose a threat to immunosuppressed patients who are at higher risk of exposure to transfusion-transmitted infections. It has been reported that a history of blood transfusion is a risk factor for toxoplasmosis [28]. Thus, the multi-transfused patient has a risk of infection with T. gondii. Therefore, there is a need to increase security measures for patients at higher risk of infection with T. gondii. Thus, multi-transfused, immunocompromised people and pregnant women should receive T. gondii-free blood. The approach could be keeping a stock of blood anti-T. gondii negative, or provision of leukoreduced (filtered) blood components, or even better, using the pathogen inactivation method [29,30].

## Conclusions

*T. gondii* infection obtained in this study was comparable to that of Algerian pregnant women and neighboring North African countries. However, this rate was lower than that recorded in other West, East, and Central African countries. Coming back to the issue highlighted at the beginning of this paper, it is now possible to state that an old infection was found in the blood donors of our region. It is well known that chronic toxoplasmosis plays a role in blood transmission as latent toxoplasmosis. It can be reactive in some circumstances and be acute in blood. In light of our results, we concluded that there is a risk of toxoplasmosis transmission through blood transfusion. To reduce the morbidity from a blood transfusion, there is a need to strengthen transfusion security measures for persons immunocompromised, multi-transfused, and pregnant women. This research appears to be the first to provide data on the seroprevalence of *T. gondii* infection among blood donors in Algeria and the risk of transmission by blood components. This report would help develop prevention strategies for transfusion-transmitted *T. gondii *infection in our country. There is a need to expand national studies, as the immune status of blood donors may vary by region due to the climatic differences in the country.
